# Responses of a Triple Mutant Defective in Three Iron Deficiency-Induced *BASIC HELIX-LOOP-HELIX* Genes of the Subgroup Ib(2) to Iron Deficiency and Salicylic Acid

**DOI:** 10.1371/journal.pone.0099234

**Published:** 2014-06-11

**Authors:** Felix Maurer, Maria Augusta Naranjo Arcos, Petra Bauer

**Affiliations:** 1 Dept. Biosciences-Plant Biology, Saarland University, Saarbrücken, Germany; 2 Institute of Botany and Cluster of Excellence on Plant Sciences (CEPLAS), Heinrich Heine University, Düsseldorf, Germany; Iwate University, Japan

## Abstract

Plants are sessile organisms that adapt to external stress by inducing molecular and physiological responses that serve to better cope with the adverse growth condition. Upon low supply of the micronutrient iron, plants actively increase the acquisition of soil iron into the root and its mobilization from internal stores. The subgroup Ib(2) *BHLH* genes function as regulators in this response, however their concrete functions are not fully understood. Here, we analyzed a triple loss of function mutant of *BHLH39*, *BHLH100* and *BHLH101* (*3xbhlh* mutant). We found that this mutant did not have any iron uptake phenotype if iron was provided. However, under iron deficiency the mutant displayed a more severe leaf chlorosis than the wild type. Microarray-based transcriptome analysis revealed that this mutant phenotype resulted in the mis-regulation of 198 genes, out of which only 15% were associated with iron deficiency regulation itself. A detailed analysis revealed potential targets of the bHLH transcription factors as well as genes reflecting an exaggerated iron deficiency response phenotype. Since the *BHLH* genes of this subgroup have been brought into the context of the plant hormone salicylic acid, we investigated whether the *3xbhlh* mutant might have been affected by this plant signaling molecule. Although a very high number of genes responded to SA, also in a differential manner between mutant and wild type, we did not find any indication for an association of the *BHLH* gene functions in SA responses upon iron deficiency. In summary, our study indicates that the bHLH subgroup Ib(2) transcription factors do not only act in iron acquisition into roots but in other aspects of the adaptation to iron deficiency in roots and leaves.

## Introduction

Iron (Fe) deficiency is among the most prevalent micronutrient deficiencies in humans. Since plants constitute the primary source of nutrients for a large part of the world’s population, the improvement of plants in terms of nutrient bioavailability is considered a priority [1]. Micronutrients like Fe are often present in an un-soluble form in the soil. Plants are able to mobilize such nutrients for uptake into the roots. Plants can also mobilize Fe from internal stores. Understanding the regulation of Fe acquisition and internal Fe utilization is of high importance for precision breeding of crops that are improved to either tolerate growth on alkaline and calcareous soils with poor Fe bio-availability or to accumulate a higher content of this micronutrient in bio-available form in the edible plant parts.

Genetic traits have been associated with micronutrient content and usage in plants, for example [2], [3]. Another trait was found in soybean as being linked to transcription factor genes encoding the soybean homologs of *BHLH38* and *BHLH39* [4]. The potential importance of these two transcription factor genes for Fe mobilization had previously been uncovered in studies on the plant model *Arabidopsis thaliana*. *BHLH38* and *BHLH39* belong to the so-called subgroup Ib(2) *BHLH* genes [5] and they are functionally redundant [6]–[8]. In fact, *BHLH38* and *BHLH39* are tandem duplicates on the chromosome, and they share similarity with two other *BHLH* genes, namely *BHLH100* and *BHLH101* [6], [9]–[11]. All these four subgroup Ib(2) *BHLH* genes are highly induced by low Fe supply in roots and leaves while they are not usually found expressed under sufficient Fe supply [6]. Expression of *BHLH39* and *BHLH101* in response to iron can be followed using the public microarray data in Arabidopsis [12]–[16] and it was found that they occur in a co-expression network along with several Fe homeostasis genes like *FERRIC REDUCTASE OXIDASE3* (*FRO3*), *NATURAL RESISTANCE-ASSOCIATED MACROPHAGE PROTEIN4* (*NRAMP4*) and *NICOTIANAMINE SYNTHASE4* (*NAS4*) [17]. From the co-expression with Fe homeostasis genes it can be concluded that the subgroup Ib(2) *BHLH* transcription factor genes likely perform regulatory functions in the context of Fe homeostasis and internal Fe mobilization. The bHLH protein POPEYE (PYE, belonging to another bHLH subgroup) is also induced by Fe deficiency within this co-expression network and it acts as a negative regulator of *FRO3*, *NRAMP4* and *NAS4,* presumably to avoid over-activation of Fe mobilization [18]. PYE is regulated by BRUTUS (BTS) that is also found in this co-expression network [17], [18]. bHLH subgroup Ib(2) can physically interact with the bHLH FER-LIKE IRON DEFICIENCY-INDUCED TRANSCRIPTION FACTOR (FIT) [8], [19]. FIT is expressed specifically in roots and has been shown to be essential for Fe uptake [20]–[23] by regulating the expression of the genes encoding ARABIDOPSIS H^+^-ATPASE2 (AHA2) [17], Fe reductase FERRIC OXIDASE2 (FRO2) [22], [24] and the IRON-REGULATED TRANSPORTER1 (IRT1) [22], [25]. From ectopic FIT expression experiments along with yeast promoter activation assays and inducible FIT activation in plants, it can be concluded that FIT targets *FRO2* and *IRT1* gene promoters [22], [23], [26], [27]. However, FIT induces *IRT1* and *FRO2* only upon Fe deficiency even when overexpressed [22], [26]. The activation of FIT at low Fe can be explained with the presence of bHLH subgroup Ib(2) factors. Indeed, the double overexpression of FIT together with either bHLH subgroup Ib(2) protein leads to an increase of Fe acquisition responses under sufficient Fe supply conditions, and it was therefore proposed that the function of bHLH subgroup Ib(2) might be to induce Fe deficiency responses in conjunction with FIT [8], [19]. However, the occurrence of *BHLH* subgroup Ib(2) genes in the *PYE* coexpression network, their non-expression upon sufficient Fe (where *FIT* and *IRT1* are active although at low level) and their high induction upon Fe deficiency not only in roots but also in leaves (in contrast to Fe acquisition genes) renders this hypothesis questionable. Moreover, contradictory results have been published with regard to the function of bHLH subgroup Ib(2) proteins. In one report, double *bhlh100 bhlh101* knockout mutants were demonstrated to develop a more severe leaf chlorosis than the wild type upon Fe deficiency, while no phenotype was apparent upon Fe sufficiency. Although some Fe homeostasis genes appeared mis-expressed, the gene knockouts did not affect the plants’ abilities for Fe uptake and the regulation of *FRO2* and *IRT1* upon sufficient or deficient Fe supply [7]. In contrast to that, in another report, bHLH subgroup Ib(2) knockouts including *bhlh100 bhlh101* and a triple knockout *bhlh39 bhlh100 bhlh101* were demonstrated to affect Fe acquisition responses and to have low *FRO2* and *IRT1* expression upon sufficient or deficient Fe supply [28]. This latter finding was rather puzzling, and it was not further explained how this finding fits to the observation that the *BHLH* genes are not normally expressed upon sufficient Fe supply, when Fe also needs to be acquired via FRO2 and IRT1 [22], [29]. Thus, the function of the bHLH subgroup Ib(2) transcription factors in Fe uptake is still open for debate.

Very interestingly, it has been shown that *BHLH38* and *BHLH39* were induced after application of salicylic acid ( = SA) by the SA-inducible Dof ( = DNA binding with one finger) transcription factor OBF BINDING PROTEIN3 (OBP3) [30]. Binding of OBP3 to promoter elements in *BHLH38* and *BHLH39* genes and their subsequent activation was demonstrated (in these studies *BHLH38* and *BHLH39* were named *OBP3 RESPONSIVE GENE2*, *ORG2*, and *OBP3 RESPONSIVE GENE3*, *ORG3*) [30]. Jasmonic acid negatively affects the onset of Fe mobilization and the induction of *FRO2* and *IRT1* [31], while ethylene enhances the responses [32]–[34]. Since SA, jasmonic acid and ethylene act in stress response networks, the possibility exists that perhaps, there is a link between SA and the up-regulation of Fe deficiency responses.

Here, we made use of the triple knockout mutant *bhlh39 bhlh100 bhlh101* (*3xbhlh*) that we constructed to investigate the functions of these *BHLH* genes in the Fe deficiency response and to further shed light on the question whether SA is involved in mediating the onset of Fe uptake via the induction of *BHLH* subgroup Ib(2) genes. We discuss that *BHLH39*, *BHLH100* and *BHLH101* are essential for a subset of Fe deficiency responses but not including up-regulation of *IRT1* and *FRO2*. We suggest that these transcription factors are involved in adapting stress responses and internal metabolic responses to Fe deficiency.

## Materials and Methods

### Plant Materials

Wild type was Col-0. The *3xbhlh* mutant line was generated from the single T-DNA insertion mutants *bhlh39-1* (SALK_025676), *bhlh100-1* (SALK_074568) and *bhlh101-1* (SALK_011245) [6]. A homozygous *bhlh39-1* plant was crossed with a *bhlh100-1 bhlh10-1* double mutant plant. In the F2 progeny a triple homozygous *bhlh39-1 bhlh100-1 bhlh101-1* plant was identified by genotyping and multiplied to obtain a triple homozygous line, hereafter named *3xbhlh*. The SA mutant line *npr1-1* (hereafter named *npr1*) with a defect in a central regulator component of SA signaling resulting in the failure of the expression of the *PR1* gene was obtained from the NASC stock center (N3726) [35]. The lines *NaHG* and *sid2-2* were provided by Fred Ausubel, Massachusetts General Hospital [36].

### Plant Growth

Arabidopsis seeds were surface-sterilized with 6% NaOCl, 0.1% Triton-X for 10 minutes, and washed 5 times with distilled water. Seeds were stratified for 2 days in 0.1% plant agar in the dark at 4°C. For the 6-day growth assay seeds were placed on Hoagland agar medium containing 50 µM FeNaEDTA (sufficient Fe supply, hereafter termed +Fe) or 0 µM FeNaEDTA (deficient Fe supply, hereafter termed −Fe), germinated and grown for 6 d under long-day conditions with 8 h dark and 16 h light [34]. On day 6, seedlings were harvested for analysis.

For the 2-week growth assay, seeds were germinated and seedlings grown for 14 d on Hoagland agar medium as described above containing 50 µM FeNaEDTA, then transferred for 3 days to fresh medium containing either 0 µM FeNaEDTA (−Fe) or 50 µM FeNaEDTA (+Fe). Then, leaves and roots were harvested separately for RNA or protein analysis. If indicated in the text 100 µM methyl-salicylic acid (hereafter named SA, Sigma-Aldrich, USA) was added to the growth medium and plants exposed for the indicated time.

### Physiological Analysis

**Figure 1:**
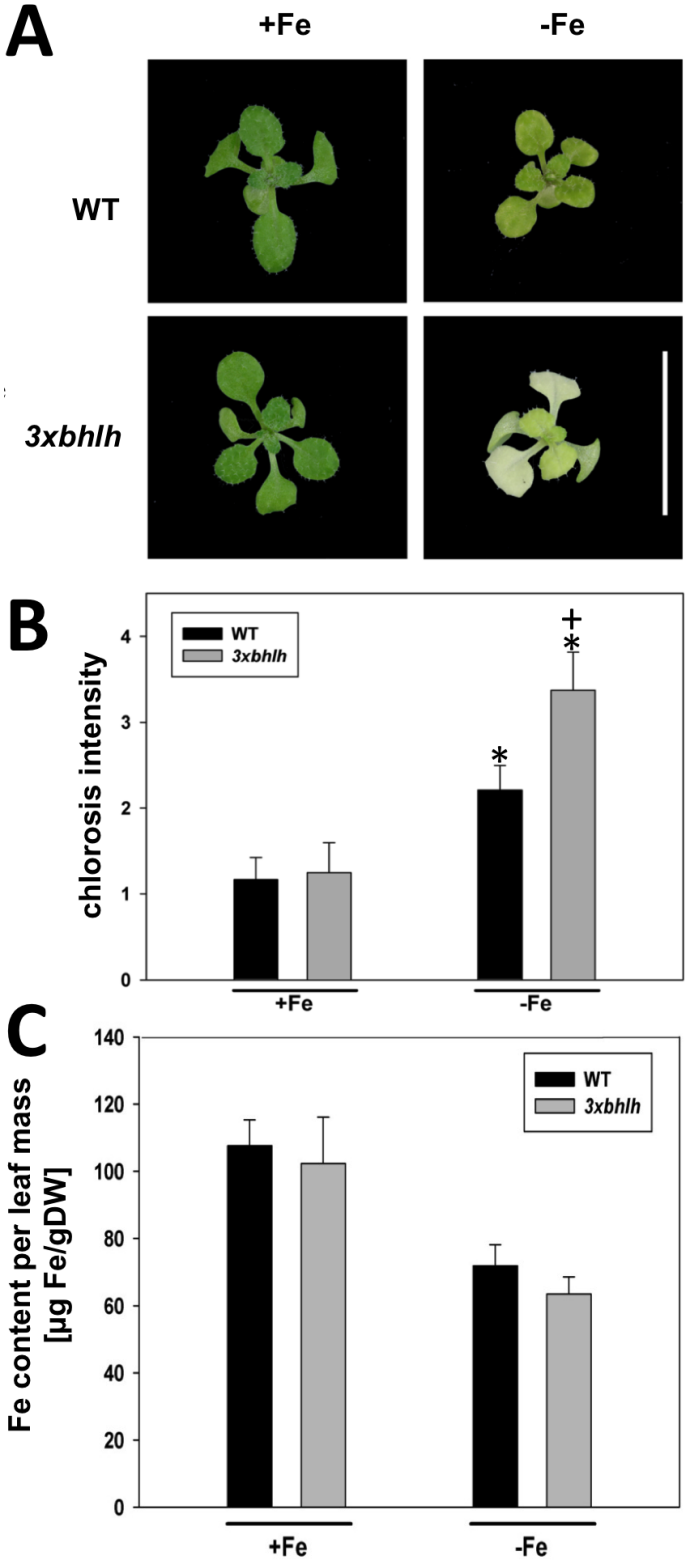
Leaf chlorosis phenotypes of the *3xbhlh* mutant. A, 10-old WT and *3xbhlh* plants grown at +Fe and −Fe; bar = 5 mm; B, Strength of leaf chlorosis; the leaf chlorosis scale used was 1 = green, 2 = green, partially yellow, 3 = yellow-green, 4 = yellow; 5 = white-yellow; n = 12; C, Fe content per leaf dry mass; n = 4; indicates a significant change (p<0.05) of −Fe versus +Fe; + indicates a significant change (p<0.05) of *3xbhlh* versus WT.

The degree of leaf chlorosis was assessed according to a previously published procedure [37]. The leaf chlorosis scale is mentionned in the figure legend of Fig. 1.

For metal determination, plant parts exposed to plant medium were washed with 100 mM Ca(NO_3_)_2_ prior to harvest to eliminate metal residues from the growth medium. Plant material was dried overnight at room temperature, then for 1 d at 120°C and powdered with an achat mortar. Quantification of metal contents of the plant samples was performed using atomic absorption spectroscopy coupled with a graphite tube atomizer as described [34]. Four technical replicate measurements were carried out with weighted samples of 50–120 µg for every atomisation (2300°C) and mean mass per dry weight values were calculated for each biological sample. Four biological replicates were produced and mean values calculated.

Iron reductase activity assays were performed using a liquid ferrozine assay [38] and biological replicates were performed as described in the figure legends. Statistical analysis was performed using the t-test.

### Gene Expression using Reverse Transcription-qPCR

Reverse transcription-quantitative real-time PCR was performed as previously described [39]. Briefly, DNase-treated RNA was used for cDNA synthesis. SYBR green I-based real-time PCR analysis was performed by using the TaKaRa Premix (TaKaRa, Japan) in the real-time ICycler (Bio-Rad, USA). For each gene, the absolute quantity of initial transcript was determined by standard curve analysis using mass standards. Absolute expression data was normalized against the averaged expression values of the internal control gene *EF1BALPHA2* [39]. Each biological cDNA sample was tested in two technical replicates and the values averaged. Statistical analysis was performed by ANOVA using the values of biological replicates. Information on oligonucleotide primer sequences is available in Table S1.

### Gene Expression using Microarray Analysis

Wild type and *3xbhlh* seedlings were grown in the 6-day growth system at –Fe, treated for six hours with 100 µM SA or were mock-treated and harvested for RNA preparation. Three biological replicates were generated. RNA was purified using the Qiagen kit and checked for integrity. Microarray hybridization was performed using the Agilent one-color gene expression V4 chip (4×44 k) for *Arabidopsis thaliana*. Microarray chip hybridization and processing were done by ATLAS Biolabs GmbH, Berlin, Germany. The obtained data were further processed, checked for quality and filtered using the GeneSpring Software, Agilent Technologies, USA, according to the GeneSpring protocol. Full microarray data are available from the NCBI site of Gene Expression Omnibus under the series GSE41774. Interesting probes were identified based on fold change analysis with a fold change cut-off of 1.5 in four pairs of conditions which were *3xbhlh* versus wild type, *3xbhlh* + SA versus wild type + SA, wild type + SA versus wild type and *3xbhlh* + SA versus *3xbhlh*. Probes were retained if they passed the moderated t-test with p<0.05. The differentially regulated probes of the four pairs of conditions were then used to construct Venn diagrams to identify groups with unique and commonly regulated probes. Probe names of these groups were converted into Arabidopsis gene ID numbers. The groups of differentially expressed genes were then further analyzed using Venn diagrams, the ATTED co-expression tool [40], the GOrilla GO annotation tool [41] and the Genevestigator tool [42].

## Results

### The *3xbhlh* Mutant was Sensitive to Fe Deficiency but not Affected in Fe Acquisition and Fe Transport to Shoots

To analyze the functions of *BHLH* subgroup Ib(2) genes, we generated a multiple loss of function mutant. The triple homozygous *bhlh39-1 bhlh100-1 bhlh101-1* knockout mutant, hereafter named *3xbhlh*, was fully fertile and did not express any full-length *BHLH39*, *BHLH100* and *BHLH101* transcripts while it expressed *BHLH38* at a higher level at Fe deficiency (hereafter termed −Fe) in roots and leaves. *BHLH38* was also found induced in the *3xbhlh* mutant roots at sufficient Fe (hereafter termed +Fe) compared to wild-type roots (Fig. S1A, B). In the same experiment, all four *BHLH* genes were highly expressed at –Fe in wild type plants, while they were not expressed or expressed at low level at +Fe (Fig. S1A, B), as expected [6]. The increased *BHLH38* expression in the *3xbhlh* mutant especially at –Fe might be due to a feedback control conferred by the triple loss of function phenotype.

**Figure 2:**
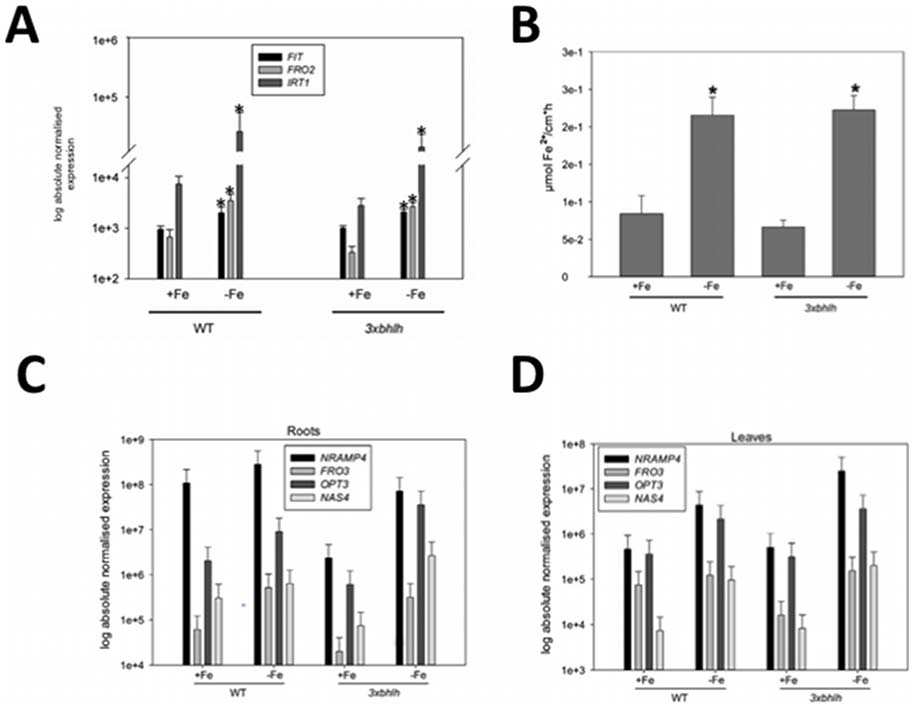
Molecular and physiological Fe acquisition responses of the *3xbhlh* mutant. A, Gene expression of *FIT*, *FRO2*, *IRT1* in roots; B, Fe reductase activity; C, *NRAMP4*, *FRO3*, *OPT3*, *NAS4*, gene expression in roots; D, *NRAMP4*, *FRO3*, *OPT3*, *NAS4*, gene expression in leaves; 14 d-old plants were transferred for three days to + and −Fe. Roots were harvested for analysis. n = 4; * indicates a significant change (p<0.05) of −Fe versus +Fe; + indicates a significant change (p<0.05) of *3xbhlh* versus WT. Gene expression was studied using reverse transcription-qPCR.

To determine whether the triple *3xbhlh* mutant had any Fe deficiency phenotype at + or –Fe, we grew *3xbhlh* and wild type plants at +Fe and –Fe. Morphological alterations in root and shoot growth were not apparent at +Fe. However, at –Fe, the *3xbhlh* mutant plants had a stronger leaf chlorosis than wild type, while root growth was normal (Fig. 1A, B). This observation suggested that *3xbhlh* mutants might be perhaps Fe deficient. This assumption was tested and could be rejected after the determination of shoot Fe contents. Neither under + nor –Fe, we could detect any differences in Fe content in the mutant versus the wild type (Fig. 1C), suggesting that the mutant was not Fe deficient. After retransfer of *3xbhlh* mutants from –Fe to +Fe the leaf chlorosis phenotype disappeared within 1–2 days (data not shown). This observation confirmed that indeed the triple *3xbhlh* mutant was able to acquire Fe. We verified this point by analyzing gene expression of Fe acquisition genes. We observed that *IRT1*, *FRO2* and *FIT* were significantly up-regulated at –Fe in wild type and *3xbhlh* plants, and that no significant difference in the expression levels between wild type and mutant was detected (Fig. 2A). A reduced expression of these Fe acquisition genes is typical for mutants affected in the regulation of Fe deficiency responses, such as the chlorotic *fit* mutant [22]. Fe reductase activity was also detected at comparable levels in mutant and wild type (Fig. 2B). An increase of Fe reductase activity was noted at –Fe but due to high standard deviations it was not found significant, but clearly was not lower than in the wild type (Fig. 2B; [22]).

In addition, we tested whether two of the PYE-regulated Fe homeostasis genes of the *PYE*/*BHLH39*/*BHLH101* co-expression network that function in internal Fe mobilization were affected in the mutant. No differences in gene expression were noted for *NRAMP4* and *FRO3* in roots and leaves (Fig. 2C, D).

Taken together, *3xbhlh* mutant plants were fully capable of regulating internal and external Fe mobilization, Fe acquisition and Fe transport genes despite of the lack of the three transcription factors. The *3xbhlh* mutant was also able to mobilize Fe from roots into the shoots. Thus, the strong leaf chlorosis of the triple mutant at –Fe cannot be the consequence of a defect in Fe acquisition and mobilization responses.

### SA and SA Signaling do not Affect Fe Deficiency Responses

**Figure 3:**
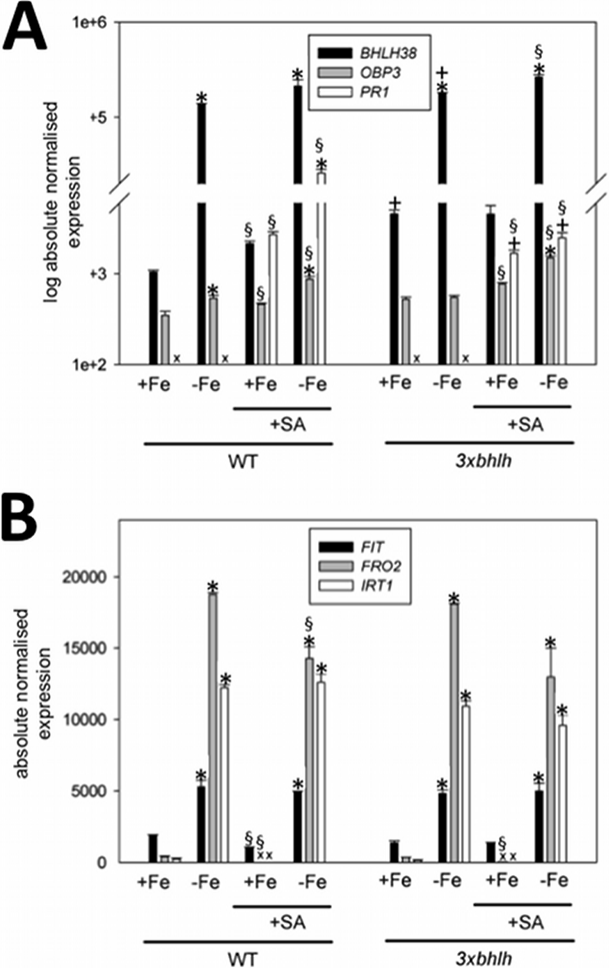
Gene expression of Fe deficiency and SA response genes in *3xbhlh* and wild type plants in response to SA and Fe. A, *BHLH38*, *OBP3*, *PR1*; B, *FIT*, *FRO2*, *IRT1*; *3xbhlh* and wild type seedlings were grown for 6 d at + and −Fe and exposed for 6 h to 100 µM SA (+SA) or were mock-treated (−SA). Whole seedlings were harvested for analysis. n = 2; * indicates a significant change (p<0.05) of −Fe versus +Fe; + indicates a significant change (p<0.05) of *3xbhlh* versus WT; § indicates a significant change (p<0.05) of +SA versus –SA. Gene expression was studied using reverse transcription-qPCR.

Since the *BHLH39*, *BHLH100* and *BHLH101* genes were dispensable for Fe uptake, but yet the plants showed a chlorosis phenotype at –Fe, we reasoned that the chlorosis phenotype could be the result of an altered adaptation to Fe deficiency stress. Since previous reports established a connection between the *BHLH* subgroup Ib(2) genes and SA signaling [30] we hypothesized that perhaps SA responses interfered with Fe deficiency regulation via the bHLH subgroup Ib(2) proteins in the process of adaptation to –Fe. To test this hypothesis, we analyzed available gene expression data of *OBP3* (At3g55370), the regulator of *BHLH38* and *BHLH39* [30]. *OBP3* is not in a co-expression network with any known Fe-regulated metal homeostasis genes. However, its expression was reported to occur in the root stele where the subgroup Ib(2) gene promoters are also active [6], [43]. SA plays a role throughout plant development and hence this could require an adaptation to Fe homeostasis [44]. To test a possible interference of SA and Fe signaling, we first tested using the seedling growth assay whether the SA response gene *PATHOGENESIS RELATED1* (*PR1*) as a marker for SA responses [35], *OBP3* and *BHLH38* were regulated by SA and Fe deficiency treatments in wild type and *3xbhlh* mutant plants. As expected, *PR1* was induced by 100 µM SA in the wild type, while *BHLH38* was induced by –Fe (Fig. 3A). These two marker genes were not per se induced by the respective other treatment, and in two out of three experiments, the expression levels were not affected in the *3xbhlh* mutant (Fig. 3A). *OBP3* was hardly induced by SA treatment and did not show any regulation by Fe or in response to the *3xbhlh* mutant. We did also not detect any differences in gene expression levels of *IRT1*, *FRO2* and *FIT* at + versus –SA treatment (Fig. 3B). Therefore, we conclude that a clear SA response did not take place upon –Fe and that the *3xbhlh* mutant did not show an altered SA response upon SA application.

**Figure 4:**
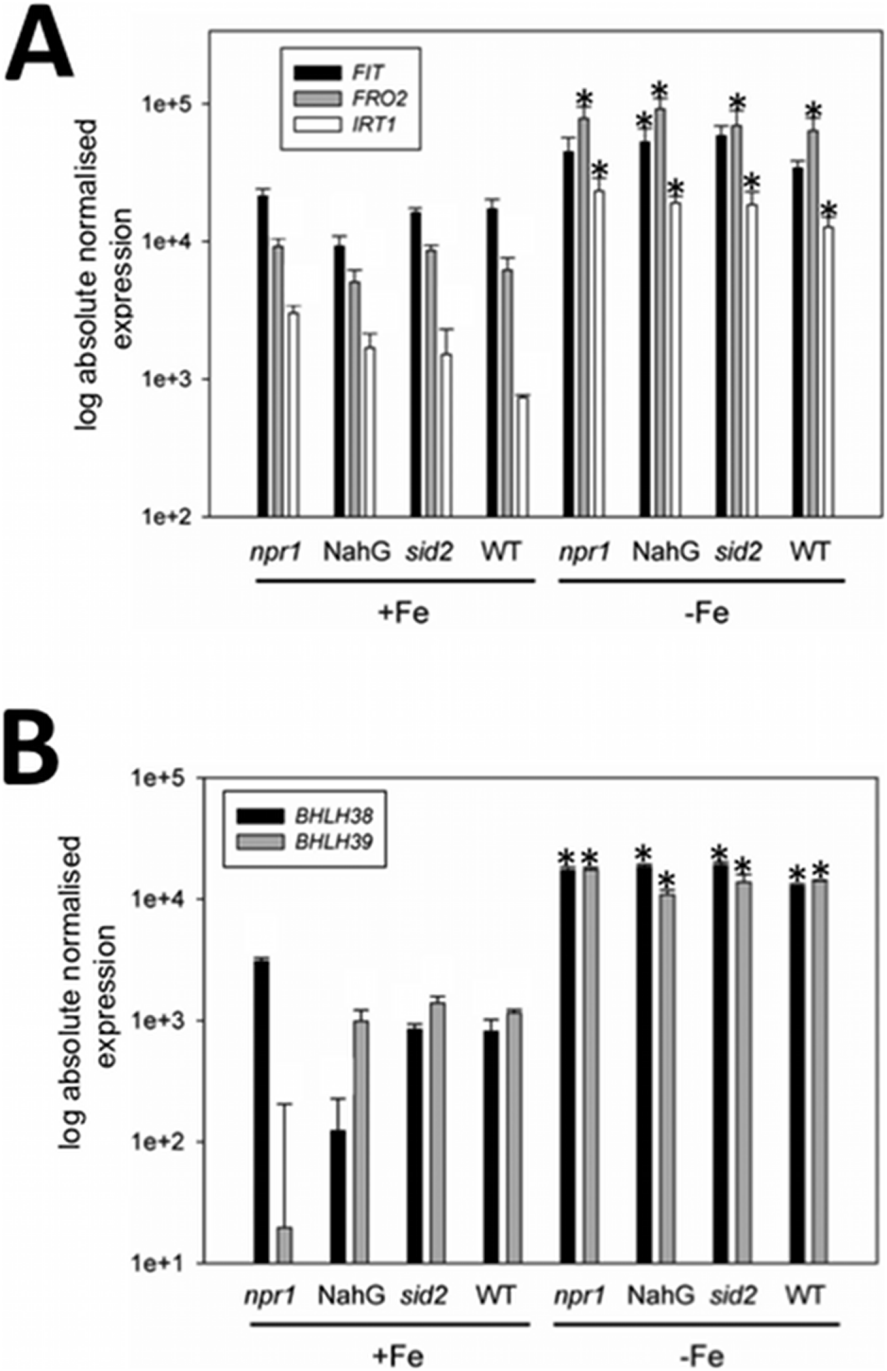
Gene expression of Fe deficiency response genes in various SA mutants and wild type plants. A, *FIT*, *FRO2*, *IRT1*; B, *BHLH38*, *BHLH39*; SA mutants and wild type seedlings were grown for 11 d at + and −Fe. Roots were harvested for analysis. n = 2; * indicates a significant change (p<0.05) of −Fe versus +Fe; + indicates a significant change (p<0.05) of SA mutant versus WT. Gene expression was studied using reverse transcription-qPCR.

Since it is possible, that SA might have a more subtle effect in the regulation of Fe deficiency responses, we also tested Fe deficiency gene regulation in various SA mutants grown under + and −Fe supply. The *non-repressor of pr1* (*npr1*) mutant is defective in SA signaling [35]. *sid2* mutant plants are defective in SA production due to lack of isochorismate synthesis, and *NahG* plants overexpress a bacterial SA hydroxylase so that SA is rapidly transformed into catechol and consequently does not accumulate [36]. We found that in these mutants Fe acquisition genes *IRT1*, *FRO2* and *FIT* were up-regulated by –Fe as in the wild type (Fig. 4A). *IRT1* was slightly up-regulated at +Fe in *npr1* compared to the wild type in one experiment. *BHLH38* and *BHLH39* expression was not affected in the mutants (Fig. 4B).

Hence, we exclude an apparent effect of SA treatment or SA signaling on Fe deficiency regulation. The up-regulation of *BHLH* subgroup Ib(2) genes under –Fe was not likely the consequence of a SA signal.

### Wild Type and *3xbhlh* Mutants Differ in Gene Expression Patterns at –Fe and in the Presence of SA

To get further hints on the Fe deficiency phenotype we performed a transcriptome comparison between *3xbhlh* and wild type seedlings (Fig. S2). This analysis was conducted under –Fe conditions since we expected most of the differential gene expression to occur at –Fe as deduced from the *3xbhlh* leaf chlorosis phenotype at –Fe. SA was included as a treatment to search for changes in gene expression in response to SA. 6-day old seedlings were grown on –Fe, transferred for 6 h to + or −100 µM SA containing –Fe medium and harvested. Microarray analysis was performed using the Agilent V4 gene chip (26.283 Arabidopsis genes). In parallel, we used the same RNA samples to perform real time quantitative reverse transcription-PCR to assess the technical quality of the method and the biological quality of the samples in this experiment (Fig. S2).

**Figure 5:**
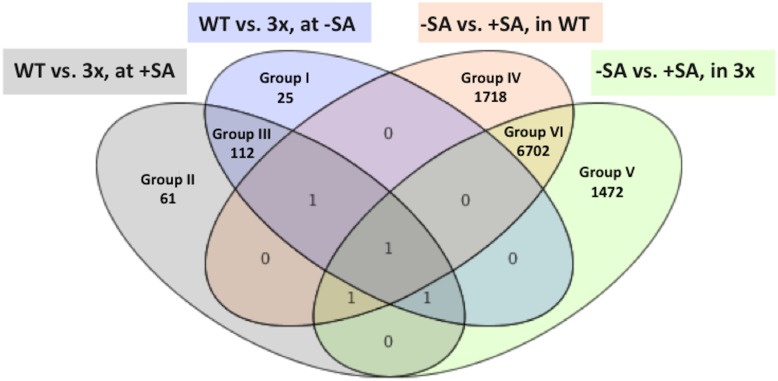
Venn diagram showing overlap of differentially regulated genes identified in microarray analysis. Four lists of genes that were differentially expressed at least 1.5-fold between the indicated conditions were used to construct the Venn diagram. The genes of groups I–V are listed in Tables S3, S4. Groups I to III contain genes differentially expressed between *3xbhlh* and wild type. Groups IV and V contain genes that show differential regulation between + and –SA treatment but not between wild type and mutant.

At first, we identified from the microarray data those genes that showed differential regulation at least 1.5-fold between wild type and the *3xbhlh* mutant in the absence and presence of SA in a statistically significant manner. The differentially regulated genes were grouped according to expression patterns using Venn diagrams (Fig. 5). 198 genes were identified as being regulated between mutant and wild type (Table S2; Fig. 5). 25 of these genes were only found deregulated in the mutant in the absence of SA (group I) and 61 genes only in the presence of SA (group II). 112 genes were regulated between mutant and wild type in the presence and absence of SA (group III). An important control for the quality of our microarray hybridization experiment was represented by the *BHLH39*, *BHLH100* and *BHLH101* genes which we found down-regulated in the *3xbhlh* mutant compared to the wild type in the group III, as was expected (Table S2). On the other hand, 9892 genes were differentially regulated between + and − SA in the wild type or in the *3xbhlh* mutant (Fig. 5). Among these latter genes 1718 were only differentially expressed in response to SA in the wild type (group IV), while 1472 other genes were specifically affected by SA in the mutant (group V) (Table S3; Fig. 5). 6702 genes were regulated by SA in wild type and in the *3xbhlh* mutant (group VI) and were not further investigated as they reflected purely SA-dependent genes. (Fig. 5). The high numbers of SA-regulated genes might indicate that the SA treatment affected the transcriptomes in a strong manner than did the *3xbhlh* mutations. However, we found that genes of groups IV and V were only mis-regulated by a maximum level of 5-fold (Table S3). On the other hand, among the small number of genes mis-regulated between mutant and wild type (groups I–III) some genes reached differential expression up to 60-fold (Table S2).

### Analysis of Functional Categories Differentially Regulated between *3xbhlh* and Wild Type Plants (Groups I to III)

Next, we assessed whether any specific functional pathways were affected in the *3xbhlh* mutant. At first, we analyzed the functions of the genes of groups I, II and III by analyzing whether specific gene ontology categories were hit among them using the GOrilla tool [41]. Among the DOWN-regulated genes of group I we found weak enrichment of categories related to lactate metabolism and cell wall, and among the UP-regulated genes of group I of categories RNA metabolism and transcription (Table S4-1, S4-2). In group II we found an enrichment for the categories flavonoid biosynthesis, UV responses, inositol metabolism and transpiration among the UP-regulated genes (Table S4-1, S4-2). In group III the categories metal response and copper binding were among the DOWN-regulated genes and again inositol metabolism among the UP-regulated genes (Table S4-1, 4-2). This analysis indicates that loss of the BHLH subgroup Ib(2) functions in the *3xbhlh* mutant at –Fe resulted in altered stress regulation and adaptation to stress.

**Figure 6:**
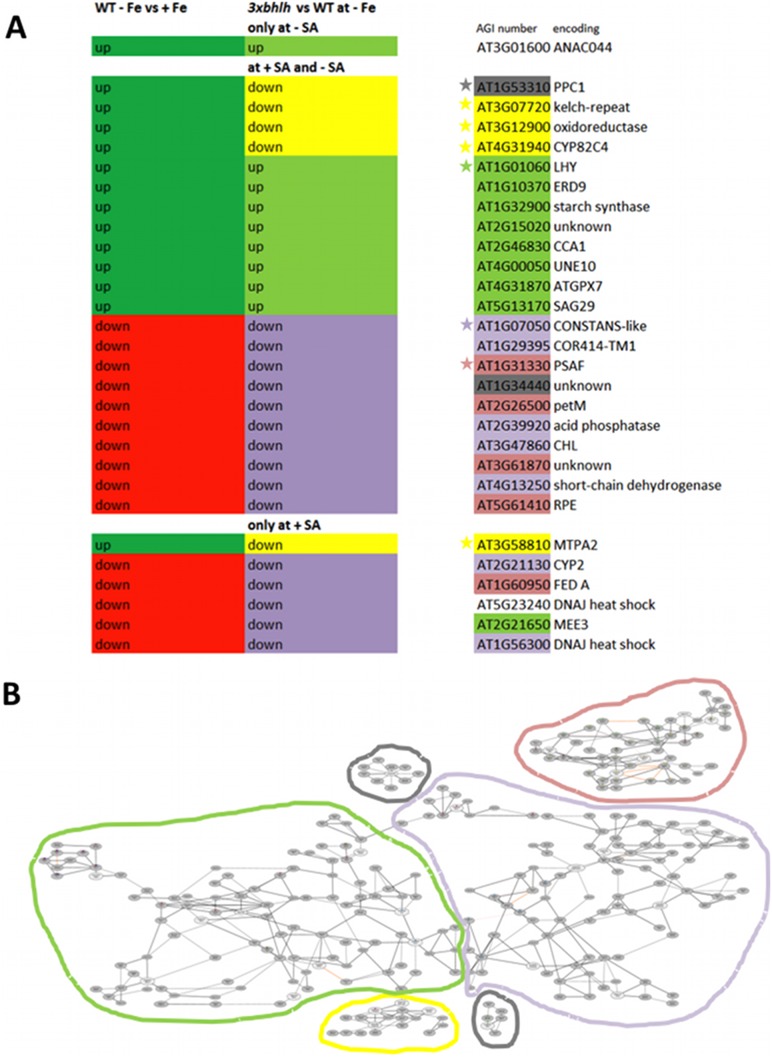
Regulation of the subset of 29 Fe-regulated genes out of groups I, II and III identified in microarray analysis. The list of Fe-regulated genes in wild type seedlings that we had published earlier [12] was used to compare with the list obtained in this work for the groups I–III. 29 genes of the groups I–III were found Fe-regulated in [12]. A, Regulation patterns, annotation and co-expression of the subset of 29 genes; the regulation at +Fe versus –Fe in the wild type is represented in the left-most column (in dark green up = up-regulated, in red down = down-regulated); the regulation in the *3xbhlh* mutant versus WT is shown in the middle column (in light green up = up-regulated and in yellow or violet down = down-regulated; note that the yellow color indicates that these genes do not follow in the *3xbhlh* mutant the regulation expected from –Fe versus +Fe in the left column and hence could be direct targets of bHLH39, bHLH100 and bHLH101). The Arabidopsis gene identification (AGI) numbers and annotations are shown on the right side, whereby the color code indicates the belonging to different co-expression networks as determined using the ATTED tool (ref), represented in B; B, Co-expression network analysis of the 29 genes; the ATTED tool was utilized for construction; the different networks are highlighted in color and the AGI numbers belonging to those networks are highlighted by the same color in A. The grey color indicates genes that are part of isolated co-expression networks. A high-resolution image of the co-expression networks is shown in Fig. S3.

Then, we checked whether the three groups I–III contained known Fe-regulated genes. In a previous work, we have grown six day-old Arabidopsis seedlings in + and −Fe conditions in the same system as utilized here. From these previous experiments we have obtained a list of iron-regulated genes in the seedlings [12], [15], [17]. The list of Fe-regulated genes in wild type seedlings published in [12] was used to compare with the list of genes in groups I–III. To our surprise we could only find 29 Fe-regulated genes among the 197 genes of the groups I–III, which corresponded to only 15% of these genes (Fig. 6A). Interestingly, these 15% of the *3xbhlh*-regulated genes could be further subdivided into 9 genes that were up-regulated by –Fe in the wild type and up-regulated in the mutant versus the wild type at –Fe as well as 15 genes that were down-regulated by –Fe in the wild type and down-regulated in the mutant versus the wild type at –Fe. Hence, the expression patterns of these 24 genes reflected the situation that the *3xbhlh* mutant was more sensitive to Fe deficiency than the wild type and that the Fe deficiency leaf chlorosis response was enhanced. Very interestingly, five genes showed an opposite expression pattern of being highly expressed at –Fe in the wild type, but expressed at low level in the *3xbhlh* mutant versus the wild type. These five genes were At1g53310 encoding PPC1, At3g07720, encoding a putative Kelch-repeat protein, At3g12900, an oxidoreductase gene, At4g31940 encoding CYP82C4 and At3g58810 encoding the metal transporter MTPA2. Therefore, it can be assumed that these five genes could represent specific target genes of the transcription factors bHLH39, bHLH100 and bHLH101. We also noted that the genes encoding the Fe acquisition machinery in Arabidopsis like *IRT1* and *FRO2* were not found among the differentially expressed genes which confirms above expression studies. We used the 29 genes to build co-expression networks using the ATTED tool [40], and in total we could identify four co-expression networks (Fig. 6B, Fig. S3). One network contained four out of the five putative bHLH targets. As mentioned above, this co-expression network was previously identified as the FIT target network [17]. Another network contained six genes up-regulated at –Fe and in the chlorotic triple mutant and one gene down-regulated in these respective conditions. This network was enriched with gene functions in flavonoid synthesis, secondary metabolism and circadian rhythm. A third network contained seven genes being down-regulated by –Fe and in the triple mutant, and this network was enriched for circadian rhythm functions. The fourth co-expression network comprised another five genes that were down-regulated at –Fe and in the *3xbhlh* mutant, and enrichment was found for photosynthetic functions and secondary metabolism. Similar functional categories were also evident for the 29 genes when using GOrilla as a GO annotation tool, namely metal response, inositol metabolism, protein folding, photosystem II and other chloroplast functions and circadian clock (Table S4-3, S4-4). In summary, co-expression analysis, functional annotation and enrichment analysis indicated that the expression of the 24 Fe-regulated genes was different in *3xbhlh* as a consequence of the increased leaf chlorosis of the triple mutant at –Fe, while the expression of five co-expressed genes indicates that they might be targets of the transcription factors in roots.

On the other hand, 85% of the genes of group I–III were not regulated by Fe deficiency. It was then interesting to determine whether these genes represented specific pathways. The functional categories were again related to inositol metabolism, circadian rhythm and UV response (Table S4-3, S4-4).

Hence, the Fe-regulated and non-Fe-regulated genes of groups I to III play functions in regulating adaptive stress processes suggesting that these functions are central for the bHLH proteins.

### Validation of Differential Gene Expression in Groups I to III by Reverse Transcription-qPCR

**Figure 7:**
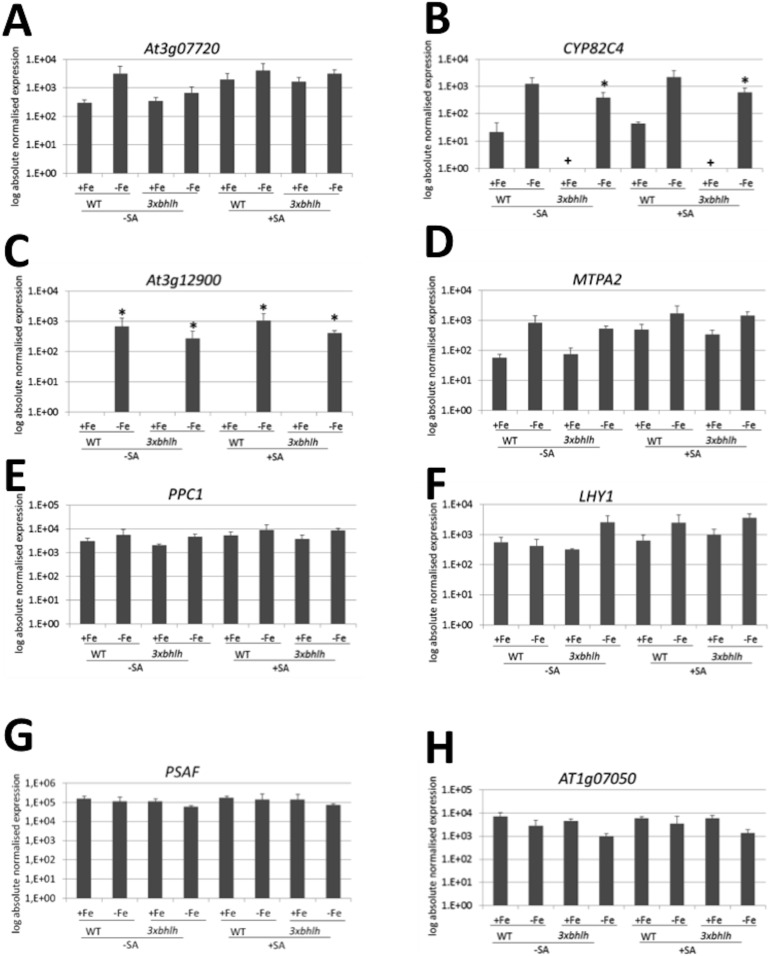
Gene expression of Fe and *3xbhlh*-regulated genes (groups I–III), identified from microarray analysis. A, At3g07720; B, *CYP82C4*; C, At3g12900; D, *MTPA2*; E, *PPC1*; F, *LHY1*; G, At1g07050; H, *PSAF*; The genes in A–E were identified as potential downstream targets of bHLH subgroup Ib(2) factors, while the genes in F–H indicated a more intense response to –Fe in the mutant (compare to Fig. 6). *3xbhlh* and wild type seedlings were grown for 6 d at + and −Fe and exposed for 6 h to 100 µM SA (+SA) or were mock-treated (−SA). Whole seedlings were harvested for analysis. n = 3; the –Fe cDNA samples were derived from the RNAs used in the microarray (Fig. S2); * indicates a significant change (p<0.05) of −Fe versus +Fe; + indicates a significant change (p<0.05) of *3xbhlh* versus WT; § indicates a significant change (p<0.05) of +SA versus –SA. Gene expression was studied using reverse transcription-qPCR.

To verify the regulatory expression patterns identified in the microarray experiments, we verified some of the gene expression results by reverse transcription-qPCR studies, especially by studying the Fe-regulated genes. Reverse transcription-qPCR was performed on the same biological samples as used for the microarray (Fig. S2). We also used the samples derived from plants grown at +Fe that we had raised in parallel. Three biological replicates have been analyzed. Due to the low number of three samples the differences were not found to be significant with p<0.05 in all expected cases, which we designated then as “tendency”. We found a tendency for up-regulation of *IRT1*, *FRO2* and *BHLH038* at –Fe versus +Fe irrespective of genotype and SA treatment which was significant for *IRT1* at −Fe versus +Fe in the *3xbhlh* mutant (Fig. S4A, B, C). *PR1* expression was significantly increased in all +SA versus –SA samples, as expected (Fig. S4D). Hence, these control gene expression results confirm that the plants had reacted as expected to Fe and salicylic acid supply in the experiment. Then, we studied gene expression of the Fe-regulated targets of the bHLH factors from groups I–III, namely of the four coexpressed genes At3g07720, *CYP82C4*, At3g12900 and *MTPA2* as well as of *PPC1*. All five genes showed a tendency to be up-regulated by –Fe in the wild type and in the *3xbhlh* mutant compared to +Fe, which was significant with p<0.05 for *CYP82C4* in *3xbhlh* and for At3g12900 (Fig. 7A–E; compare to Fig. 6, yellow co-expression network and *PPC1*). The expression of these five genes had a tendency to be lower in the mutant than in the wild type at –Fe. In the presence of SA the five genes were similarly regulated than in the absence of SA, whereby the basal expression levels at +Fe in the wild type were found higher in tendency at +SA than at −SA for At3g07720 and *MTPA2*.

Next, we verified gene expression of the Fe-regulated group III genes, namely *LHY1*, *PSAF* and At1g07050 (Fig. 7F–H; compare to Fig. 6, green, violet and pink coexpression networks, respectively). We found that *LHY1* followed a tendency to be more expressed at –Fe versus +Fe in the mutant and in the presence of SA. At1g07050 and *PSAF* followed a tendency to be expressed at lower level at –Fe versus +Fe, especially in the *3xbhlh* mutant.

Taken together, the reverse transcription-qPCR data confirmed in their tendency the gene regulation changes detected in the comparative transcriptome analysis. This result underlined the technical and biological reproducibility, however the number of only three biological replicates did not allow obtention of p<0.05 statistical values in each comparison.

### Analysis of Functional Categories Differentially Regulated between + and −SA (Groups IV and V)

Different genes were regulated by SA in the wild type (group IV) and *3xbhlh* plants (group V). We used again the GOrilla tool [41] to analyze whether specific categories were hit in the two cases. In group IV we identified the categories RNA and post-transcriptional genes silencing, organelle organization, GTP activity and chloroplast functions among the DOWN-regulated genes in the wild type, while the categories lipid metabolism, cellular starvation response and transition metal transport were hit among the UP-regulated genes (Table S4-1, 4-2). In group V the categories DNA replication, transmembrane receptor signaling, cell division, histone modification, protein binding and chloroplast thylakoid were hit among the DOWN-regulated genes and defense and heat response, signaling, kinase activity and ADP binding were identified as pathways among the UP-regulated genes (Table S4-1, 4-2).

Hence from the high number of regulated genes and the categories we deduce that SA affects stress responses and cell division in a different manner in the wild type and the *3xbhlh* mutant upon SA treatment. Thus, the *3xbhlh* mutant is in a different manner sensitive to SA than the wild type.

## Discussion

In this report we show that *BHLH* subgroup Ib(2) genes *BHLH39*, *BHLH100* and *BHLH101* acted in the adaptation to the stress caused by the Fe deficiency but not for Fe acquisition itself. Although *BHLH38* and *BHLH39* may act in the SA pathway we did not find any evidence that SA responses interfered with Fe deficiency regulation via the *BHLH* genes.

Contradictory reports in the literature rendered it difficult to establish a clear function for *BHLH* subgroup Ib(2) genes in the regulation of Fe deficiency responses in Arabidopsis. On one side, it was shown that a triple mutant *bhlh39 bhlh100 bhlh101* was affected in the ability to up-regulate Fe reductase activity upon –Fe [28], while a double mutant *bhlh100 bhlh101* was not [7]. However, in both these studies the mutants were described to display a leaf chlorosis at –Fe but not at +Fe. Here we demonstrate that the *3xbhlh* triple mutant *bhlh39 bhlh100 bhlh101* developed a leaf chlorosis only at –Fe but not at +Fe. We found and confirmed that this phenotype was clearly not associated with a reduced uptake of Fe.

When we analyzed the triple *3xbhlh* mutant we observed that this mutant was able to mobilize and acquire Fe, in contrast to the report mentioned above [28]. The first evidence came from the fact that the leaf chlorosis was restrained to –Fe, while no such phenotype was observed at +Fe. The *3xbhlh* mutants had similar Fe levels as the wild type, and hence, these plants were able to utilize Fe at +Fe like the wild type. In addition, the *3xbhlh* mutants were able to induce *FIT*, *IRT1* and *FRO2* at –Fe versus +Fe. The three Fe uptake genes did also not occur among the groups I–III that comprised the genes differentially expressed between mutant and wild type. Fe reductase activity was clearly inducible in the triple mutant at least to the same extent as in the wild type. An alternative possibility to explain the increased leaf chlorosis of *3xbhlh* mutant compared to wild type plants at –Fe could be linked with a reduced ability of the mutant to utilise internal iron. However, we did not find evidence that typical genes for internal Fe utilization were changed in their expression, like *NRAMP4* [45], *FRO3* [46], *OPT3* [47] and *NAS4* [48], [49]. Taken together, we can say that the leaf chlorosis of *3xbhlh* plants was not the consequence of impaired Fe uptake and utilisation. Since *BHLH38* was still expressed in the *3xbhlh* mutant, even to a higher level than in the wild type, we cannot conclude that none of the subgroup Ib(2) *BHLH* genes are required for Fe uptake or Fe mislocalization. Since *BHLH38* is hardly expressed at +Fe (when Fe is available for uptake), it is not likely required for Fe uptake, either. Due to the tandem location of *BHLH38* and *BHLH39* in the genome, a quadruple insertion mutant with a full knockout of all *BHLH* functions cannot be readily generated to confirm this. We predict that a quadruple mutant would have more severe leaf chlorosis symptoms than the wild type at –Fe.

The question remains what is the cause of the increased leaf chlorosis of *3xbhlh* mutants at –Fe. One clue to that question could come from the functions of the genes that we found differentially expressed in the mutant versus the wild type.

Five genes were regulated in an opposite manner in the *3xbhlh* mutant than in the wild type in response to iron. These genes might be targets for bHLH39, bHLH100 and bHLH101, namely *PPC1*, At3g07720, the oxidoreductase At3g12900, *CYP82C4* and *MTPA2*. The latter four are coexpressed with target genes of the transcription factor FIT, such as *IRT1*. However, some of the FIT targets of the co-expression network were found here not be mis-regulated in the *3xbhlh* mutant. This result can be explained. We suggest that the four bHLH transcription factors of the subgroup Ib(2) show functional divergence. bHLH38 in conjunction with FIT namely acts on the induction of the targets *IRT1* and *FRO2*, while bHLH39, bHLH100 and bHLH101 presumably together with FIT more specifically target the other genes in this co-expression network that we have found here.

Another set of Fe-regulated genes of groups I–III, which are differentially expressed in the mutant versus the wild type, indicated that the Fe deficiency responses were augmented in the mutant. For example, genes which are up-regulated at –Fe versus +Fe in the wild type showed an exaggerated up-regulation response in the comparison of mutant versus wild type at –Fe. Or, genes which are down-regulated at –Fe versus +Fe in the wild type showed a stronger down-regulation response in the mutant versus wild type at –Fe. From these expression patterns it is unlikely that these genes are regulated directly by the bHLH subgroup Ib proteins since otherwise we would have expected opposite trends of regulation between +/−Fe and mutant/wild type. The differential regulation of these genes is rather a pleiotropic effect due to the *3xbHLH* mutations. Since the most obvious phenotype was the increased leaf chlorosis, the differential regulation of these genes was likely an effect of the severe leaf chlorosis. Indeed, several of these genes have functions in the leaves related to the clock and in chloroplasts. Previous reports have established an effect of iron deficiency on the biological clock [50]–[52].

On the other hand, 170 genes were differentially expressed between mutant and wild type without that any differential expression of these genes had been noted in response to Fe supply in previous experiments [12]. Therefore, we deduce that the three *BHLH* genes regulate an adaptation response to the stress caused by the Fe deficiency. This would also explain why these *BHLH* genes are only activated at –Fe, but not at +Fe, and why they are induced in leaves and in roots. And in addition, it explains that in the absence of the genes in the *3xbhlh* mutant such a high number of genes are differentially expressed whereby in the wild type the expression appears unchanged. A further question is what could be the function of the 170 genes not regulated by Fe supply. One possibility could have been that these 170 genes are regulated by salicylic acid, a stress hormone. *BHLH38* and *BHLH39* have been brought into the context of SA regulation [30], and as a stress hormone SA signaling could be effective upon Fe deficiency stress. However, we exclude that the 170 genes are SA-regulated since none of them was in the intersections with the 50 times higher number of SA-regulated genes in the Venn diagrams, as revealed in our microarray experiments. The main functional categories that these 170 genes belong to are inositol metabolism, circadian rhythm again and UV responses. Hence, it can be concluded that the bhlh functions are important for adapting leaf responses to Fe deficiency stress. The absence of proper regulation would thus result in a more increased leaf chlorosis due to improper adaptation. Since the bHLH genes of the subgroup Ib(2) are expressed in leaves and there induced by Fe deficiency, it is plausible to predict that the leaf regulation is the main function of the bHLH genes which takes place independent of FIT.

In conclusion, it will be very interesting in the future to study the connection between leaf responses as an adaptation to Fe deficiency conferred by bHLH subgroup Ib(2) proteins.

## Supporting Information

Figure S1 Gene expression of *BHLH* subgroup Ib genes in *3xbhlh* and wild type plants in response to Fe. A, in roots; B, in leaves; *3xbhlh* and wild type seedlings were grown for 14 d at +Fe and exposed for 3 d to + or −Fe. Roots and leaves were harvested for analysis. n = 4; * indicates a significant change (p<0.05) of −Fe versus +Fe; + indicates a significant change (p<0.05) of *3xbhlh* versus WT. Gene expression was studied by reverse transcription-qPCR.
(TIFF)

Figure S2 Overview of one biological replicate set of microarray and reverse transcription-qPCR experiments; *3xbhlh* and wild type seedlings were grown for 6 d at + and −Fe and exposed for 6 h to 100 µM SA (+SA) or were mock-treated (−SA). RNA and cDNA was prepared from all samples for use in reverse transcription-qPCR analysis (see Fig. 7). Microarray analysis was only conducted with –Fe samples (see Fig. 5, 6). For reverse transcription-PCR and microarray analysis a total of three biological replicates was performed.
(TIFF)

Figure S3 High-resolution image of the co-expression network analysis of the 29 Fe-regulated genes out of groups I, II and III. The ATTED tool was utilized for construction. Further analysis and additional information are provided in Fig. 6.
(TIF)

Figure S4 Gene expression of Fe deficiency and SA marker genes in the samples used for microarray analysis. A, *FIT*; B, *IRT1*; C, *FRO2*; D, *PR1*; *3xbhlh* and wild type seedlings were grown for 6 d at + and −Fe and exposed for 6 h to 100 µM SA (+SA) or were mock-treated (−SA). Whole seedlings were harvested for analysis. n = 3; the –Fe cDNA samples were derived from the RNAs used in the microarray (Fig. S2); * indicates a significant change (p<0.05) of −Fe versus +Fe; + indicates a significant change (p<0.05) of *3xbhlh* versus WT; § indicates a significant change (p<0.05) of +SA versus –SA. Gene expression was studied using reverse transcription-qPCR.
(TIF)

Table S1 Primer sequences.
(DOT)

Table S2 List of genes of the groups I, II and III with differential expression between *3xbhlh* and wild type at –Fe in the presence and absence of 100 µM SA.
(XLS)

Table S3 List of genes of the groups IV and V with differential expression of either *3xbhlh* or wild type at –Fe in the presence and absence of 100 µM SA.
(XLS)

Table S4 GO annotation of the differentially regulated genes of groups I to V.
(XLS)
