# Supplementary figures and images for: Responses of a Triple Mutant Defective in Three Iron Deficiency-Induced BASIC HELIX-LOOP-HELIX Genes of the Subgroup Ib(2) to Iron Deficiency and Salicylic Acid
Source: PLoS One. 2014 Jun 11;9(6):e99234. doi: 10.1371/journal.pone.0099234 (PMC4053374; doi:10.1371/journal.pone.0099234)

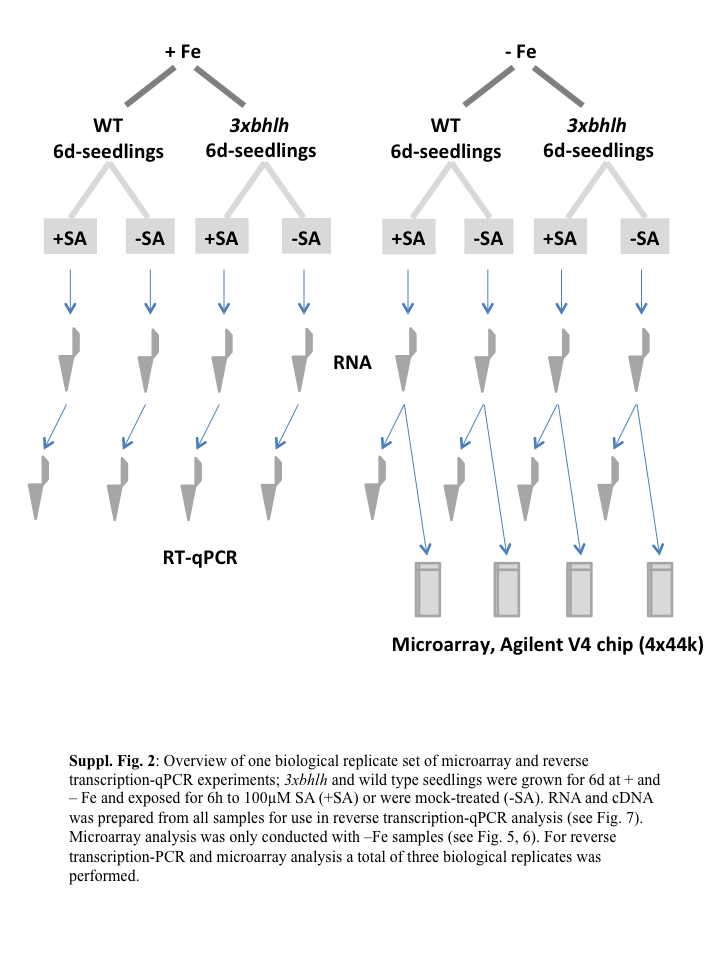

Supplement: Figure S2 — Overview of one biological replicate set of microarray and reverse transcription-qPCR experiments; 3xbhlh and wild type seedlings were grown for 6 d at + and −Fe and exposed for 6 h to 100 µM SA (+SA) or were mock-treated (−SA). RNA and cDNA was prepared from all samples for use in reverse transcription-qPCR analysis (see Fig. 7). Microarray analysis was only conducted with –Fe samples (see Fig. 5, 6). For reverse transcription-PCR and microarray analysis a total of three biological replicates was performed. (TIFF) [file pone.0099234.s002.tiff]

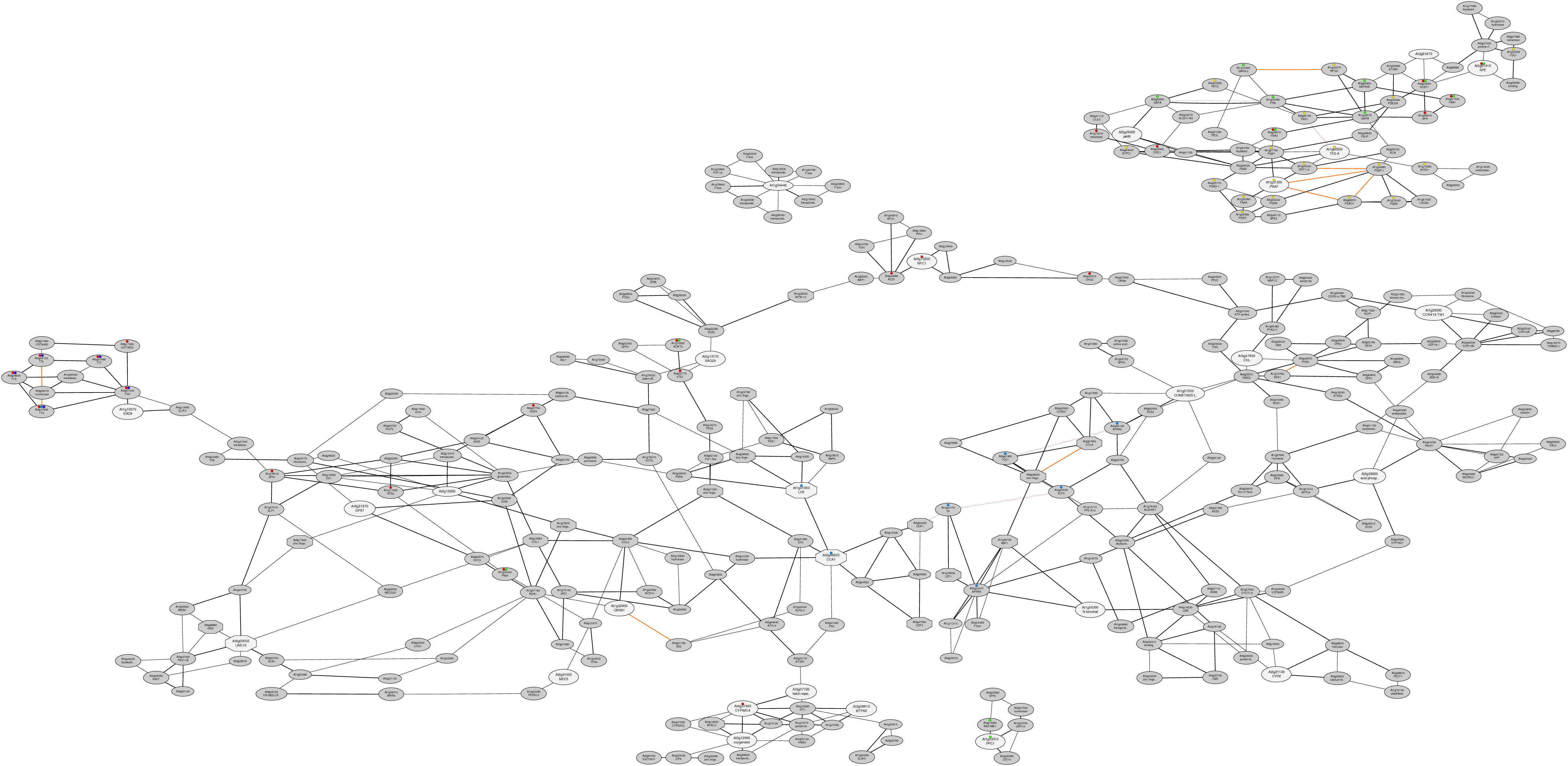

Supplement: Figure S3 — High-resolution image of the co-expression network analysis of the 29 Fe-regulated genes out of groups I, II and III. The ATTED tool was utilized for construction. Further analysis and additional information are provided in Fig. 6. (TIF) [file pone.0099234.s003.tif]

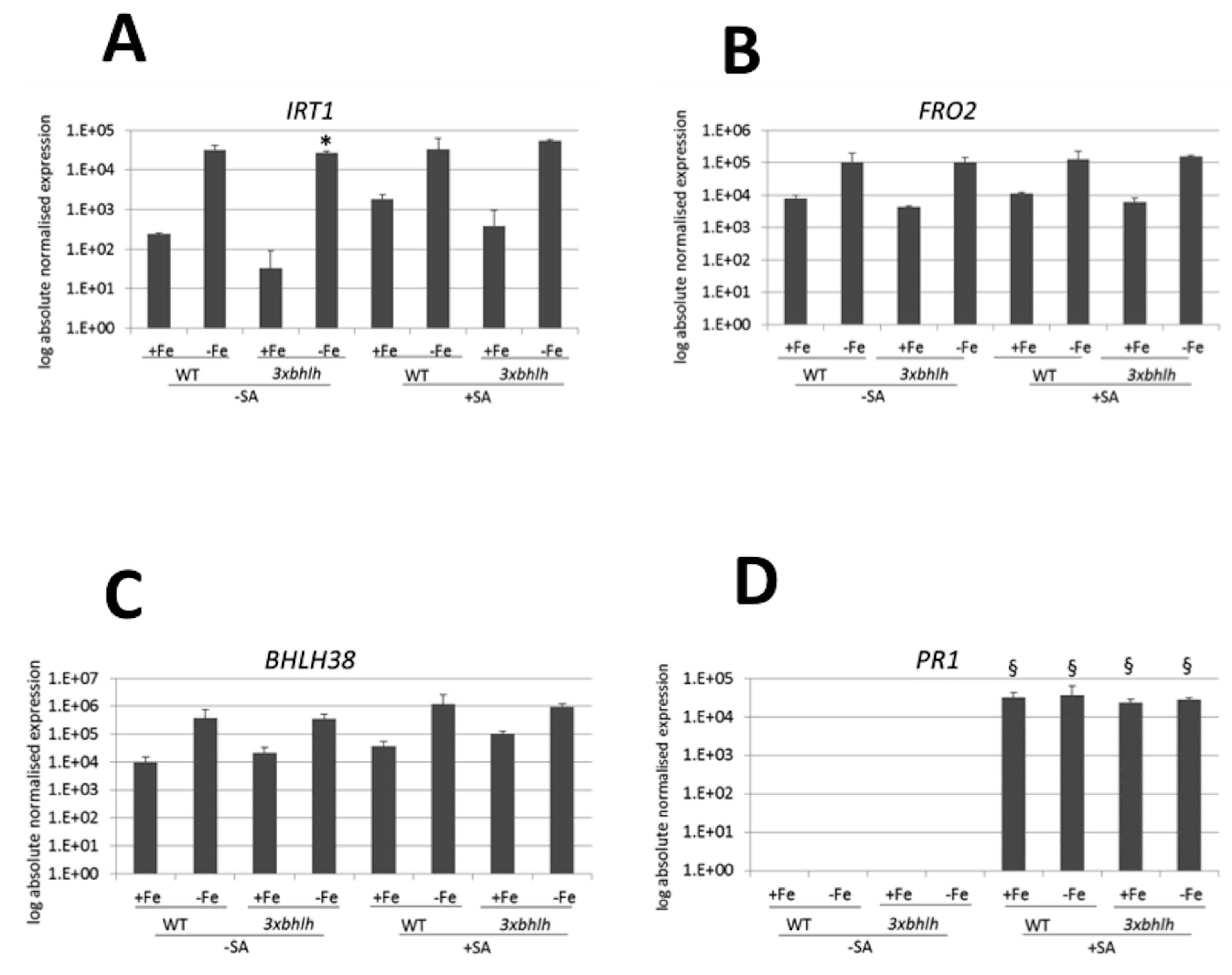

Supplement: Figure S4 — Gene expression of Fe deficiency and SA marker genes in the samples used for microarray analysis. A, FIT; B, IRT1; C, FRO2; D, PR1; 3xbhlh and wild type seedlings were grown for 6 d at + and −Fe and exposed for 6 h to 100 µM SA (+SA) or were mock-treated (−SA). Whole seedlings were harvested for analysis. n = 3; the –Fe cDNA samples were derived from the RNAs used in the microarray (Fig. S2); * indicates a significant change (p<0.05) of −Fe versus +Fe; + indicates a significant change (p<0.05) of 3xbhlh versus WT; § indicates a significant change (p<0.05) of +SA versus –SA. Gene expression was studied using reverse transcription-qPCR. (TIF) [file pone.0099234.s004.tif]
