# Supplementary material for: Responses of a Triple Mutant Defective in Three Iron Deficiency-Induced BASIC HELIX-LOOP-HELIX Genes of the Subgroup Ib(2) to Iron Deficiency and Salicylic Acid
Source: PLoS One. 2014 Jun 11;9(6):e99234. doi: 10.1371/journal.pone.0099234 (PMC4053374; doi:10.1371/journal.pone.0099234)
Supplement: Table S1 — Primer sequences. (DOT) [file pone.0099234.s005.dot]

| **Suppl. Table 1**: Oligonucleotid primers used in reverse transcription-qPCR experiments | | |
| --- | --- | --- |
| Name | AGI number | Sequence (5‘ to 3‘) |
| PR1 RT 5’ | At2g14610 | AAGTCAGTGAGACTCGGATG |
| PR1 RT 3’ | At2g14610 | GTATGGCTTCTCGTTCACAT |
| OBP3 RT 5’ | At3g55370 | ATCACCACCGACTTCTCAGG |
| OBP3 RT 3’ | At3g55370 | AGATTGCACCAATCCGGTAG |
| At3g07720 RT 5‘ | At3g07720 | TCGACCTCGAAACTCAGACA |
| At3g07720 RT 3‘ | At3g07720 | AACTCGTTGAGCTCCTGGTG |
| CYP82C4 RT5‘ | At4g31940 | AGAATGGCGGTACAAAACCA |
| CYP82C4 RT3‘ | At4g31940 | ATCCTCCGACGATACTGAGC |
| MTP3 RT5‘ | At3g58810 | CAGATGCCAAGACCAGTTCA |
| MTP RT3‘ | At3g58810 | GCAAGACTGTTGGCCTTGAT |
| At3g12900 RT5‘ | At3g12900 | ATCGTCGGCTCATGAGTTTT |
| At3g12900 RT3‘ | At3g12900 | TTCCACTCAATCGCCTTCTC |
| PPC1 RT5‘ | At1g53310 | GTTCCCTCCGTGTCACAGTT |
| PPC1 RT3‘ | At1g53310 | CTGGTTTAGGCGAAATTGGA |
| LHY1 RT5‘ | At1g01060 | GGCATTTCCACGCAGATATT |
| LHY1 RT3‘ | At1g01060 | TGCCCGTGAGTTTCTTCTCT |
| PSAF RT5‘ | At1g31330 | TCTCGTTCTCAACCCGAGAT |
| PSAF RT3‘ | At1g31330 | AGGTGTGGAGGAGGAGGATT |
| At1g07050 RT5’ | At1g07050 | CGAGAGCATTTTCGACATCA |
| At1g07050 RT3’ | At1g07050 | TCCCAAAACCCAAAACTTGA |
| SLP1 RT5’ | At1g07010 | ACCATGTTGCATACGGCATA |
| SLP1 RT3’ | At1g07010 | ATCCTCGAGTCGCAATGAAC |
| At4g14330 RT5‘ | At4g14330 | TGCTTCTGCTGTGATTTTGG |
| At4g14330 RT3‘ | At4g14330 | AGCTGCTTTTGTGCCTCATT |
| At3g59350 RT5’ | At3g59350 | CAGCGGAACCTGAATCAAAT |
| At3g59350 RT3’ | At3g59350 | ATACGCAAGAATGCGGAAAT |
| At1g15940 RT5’ | At1g15940 | AAAGGTGCACTGAAGGCTGT |
| At1g15940 RT3’ | At1g15940 | TTTTGCCAGTTCTGCCTCTT |
| P4H5 RT5’ | At2g17720 | CACTACCAAGTTGGGCAGAAG |
| P4H5 RT3’ | At2g17720 | ACAGTCTCGCCACCATCATC |
| These oligonucleotide primers were designed for this work. | | |
